# Effect of a transtheoretical model-based intervention and motivational interviewing on hyperphosphatemia management via telehealth (TMT program) among hemodialysis patients during the COVID-19 pandemic

**DOI:** 10.3389/fpubh.2024.1361778

**Published:** 2024-11-20

**Authors:** Arrom Thongsunti, Chatchawan Silpakit, Thanapoom Rattananupong, Wonngarm Kittanamongkolchai, Warangkana Sumethpimolchai, Vitool Lohsoonthorn

**Affiliations:** ^1^Health Research and Management Program, Department of Preventive and Social Medicine, Faculty of Medicine, Chulalongkorn University, Bangkok, Thailand; ^2^Department of Psychiatry, Faculty of Medicine, Ramathibodi Hospital, Mahidol University, Bangkok, Thailand; ^3^Department of Preventive and Social Medicine, Faculty of Medicine, Chulalongkorn University, Bangkok, Thailand; ^4^Division of Nephrology, Department of Medicine, Faculty of Medicine, Chulalongkorn University, Bangkok, Thailand; ^5^Maha Chakri Sirindhorn Clinical Research Center, Faculty of Medicine, Chulalongkorn University, Bangkok, Thailand; ^6^Renal Immunology and Transplantation Research Unit, Faculty of Medicine, Chulalongkorn University, Bangkok, Thailand; ^7^Hemodialysis Unit, Dontoom Hospital, Nakhon Pathom, Thailand

**Keywords:** hyperphosphatemia, transtheoretical model, motivational interviewing, telehealth, hemodialysis

## Abstract

**Background:**

Hyperphosphatemia poses a significant risk for cardiovascular diseases and mortality in hemodialysis patients. Non-adherence to phosphate binders and a low-phosphate diet behavior contribute to this issue. Leveraging psychological and behavior change theories has proven effective in addressing many health risks. During the COVID-19 pandemic, face-to-face communication was limited, and telehealth served as a bridge to address healthcare gaps. This study aimed to determine the effect of a transtheoretical model-based intervention and motivational interviewing on hyperphosphatemia management via telehealth (TMT program) among hemodialysis patients during the COVID-19 pandemic.

**Method:**

A two-arm parallel randomized controlled trial with assessors blinding involved 80 participants who were stratified block-randomized into either the TMT program group (*n* = 40) or the control group (Usual care; *n* = 40). Linear regression was used to compare the two groups on serum phosphorus levels, knowledge of hyperphosphatemia management, and dietary consumption behavior at the 24-week endpoint. The readiness to change (stage of change), self-efficacy, and phosphate binder adherence were assessed using Fisher’s test.

**Result:**

The TMT program demonstrated a significant reduction in serum phosphorus levels compared to usual care (mean difference = −1.03, 95% CI = −1.77, −0.29). Additionally, improvement in dietary consumption behavior related to phosphorus-containing foods was also observed (mean difference = 13.48, 95% CI = 8.41, 18.57). Positive effects emerged in the readiness to change (*p* < 0.001), self-efficacy in the appropriate use of phosphate binders (*p* = 0.025), and adherence to phosphate binders (*p* = 0.001) at the 24-week endpoint. However, groups did not differ in knowledge of hyperphosphatemia management (mean difference = 7.02, 95% CI = −1.03, 15.07).

**Conclusion:**

The study demonstrated that the TMT program has positive effects on reducing serum phosphorus levels, providing a hyperphosphatemia management strategy for ESRD patients undergoing hemodialysis via telehealth.

**Clinical trial registration:**

TCTR20230628003, https://www.thaiclinicaltrials.org.

## 1 Introduction

Hyperphosphatemia is a major concern for patients with end-stage renal disease (ESRD) (1, 2). It significantly contributes to chronic kidney disease mineral and bone disorder (CKD-MBD), soft tissue, cardiovascular diseases, and an approximately 2-fold increase in mortality and sudden death (3–7). The kidneys play a crucial role in maintaining phosphate homeostasis. Proper phosphate balance in the body requires adequate dietary intake, efficient renal phosphate excretion, and a regulated equilibrium between bone formation and resorption (8, 9). In patients requiring hemodialysis, impaired kidney function disrupts the ability to manage phosphate levels effectively. A combination of adequate dialysis, dietary phosphate control, and adherence to phosphate binders is essential (7–12) to reduce elevated serum phosphorus levels to the normal range of 3.5 to 5.5 milligrams per deciliter (mg/dL) (3, 13).

The inefficiency of conventional hemodialysis (3 times/week; 4 hours/session) hampers the reduction of serum phosphorus levels (6, 11). Despite removing about 2,100–3,000 milligrams of phosphorus per week through conventional thrice-weekly hemodialysis sessions, dietary phosphate restriction remains complex, as the daily intake averages around 5,000–7,000 milligrams per week (approximately 800 to 1,000 milligrams per day) (12, 14). Controlling this requires balancing protein intake and identifying phosphorus-containing food sources (11, 15–17). Patients’ lack of knowledge about the bioavailability and absorption of inorganic and organic phosphorus in food poses challenges for effective dietary control (6, 18). Additionally, the high pill burden, side effects and complex schedule affect adherence to phosphate binders (11, 19). A systematic review from 1970 to 2014 found that non-adherence among patients ranged from 13.9 to 98.6%, with an average of 52.2% (19). All these factors contribute to the difficulty of controlling serum phosphorus levels.

Moreover, a previous study revealed that merely providing patients with health education about a low-phosphate diet and phosphate binder intake is ineffective in significantly reducing serum phosphorus levels during maintenance hemodialysis (20). Chronic kidney disease (CKD) symptoms and prolonged treatment can cause patients physical, emotional, and spiritual suffering (21, 22). This suffering can elicit emotions such as despair, resignation, or fear of judgment, which affect patients’ adherence to treatment plans. Applying theories related to behavior change and communication can help patients understand their behaviors to resolve health problems. Such an approach should also improve the patient’s understanding of the disease and the need for continuous adherence to the treatment plan and inspire them to change their behaviors.

Numerous studies have employed the transtheoretical model, which identifies five stages for behavior change and recognizes the variability in individuals’ readiness to change. This model has successfully addressed health risks (23–26), including hyperphosphatemia in CKD (27–29). Effective communication, essential for providing support and motivation, is pivotal in addressing these challenges. Motivational interviewing supports a patient-centered and empathetic approach using a collaborative, goal-oriented communication style. It encourages individuals to confront ambivalence, focusing on the language of change and meaningful behavioral shifts (18, 30, 31). This approach can help promote adherence to treatment guidelines and improve clinical outcomes (31–33).

The COVID-19 pandemic exacerbated the problem by negatively affecting economic circumstances and individual consumption behaviors and limiting face-to-face communication in usual care. This also impacted applying the transtheoretical model and using motivational interviewing to promote adherence to treatment guidelines among patients. Although patients still needed to travel to the hospital for dialysis 2–3 times a week, social distancing had to be maintained between patients and medical personnel. This requirement to prevent the spread of COVID-19 made traditional individual and group counseling impractical. Healthcare professionals adapted by delivering knowledge and offering personalized dietary recommendations and counseling through telehealth, including telephone consultations and online platforms. Several studies suggest that telehealth can enhance patients’ self-care knowledge, boost self-efficacy, improve overall quality of life, and effectively address various health-related concerns (34–38).

This study evaluated the effectiveness of a newly developed Transtheoretical Model and Motivational Interviewing via Telehealth (TMT) program for managing hyperphosphatemia. The evaluation focused on serum phosphorus levels, knowledge of hyperphosphatemia management, dietary phosphorus consumption behavior, phosphate binder adherence, and readiness to change among ESRD patients undergoing hemodialysis during the COVID-19 pandemic. It also examined the use of telehealth to integrate behavior change strategies into patient care for chronic diseases, with implications for future applications.

## 2 Materials and methods

### 2.1 Research design

The present study was a randomized controlled trial (RCT), a 2-arm, parallel, single-blinded trial with blinded assessors. The trial received approval from the Institutional Review Board of the Faculty of Medicine, Chulalongkorn University (IRB No. 1005/64, Date of approval: March 3, 2022) and registered with the Thai Clinical Trials Registry (TCTR20230628003).

### 2.2 Participants

Eligible participants were ESRD patients undergoing hemodialysis at three hemodialysis centers located in Chachoengsao and Nakhon Pathom Provinces. Two of these centers are in community hospitals; the remaining center is affiliated with a general hospital. These provinces are in the eastern and central regions near the Bangkok metropolis, Thailand. The eligibility criteria were as follows: (1) Age 18 or older and a minimum of 3 months of hemodialysis (2) an average serum phosphorus of ≥5.5 mg/dL over 3 months, (3) adequate hemodialysis measured by a Kt/V value ≥1.8 for patients undergoing hemodialysis twice a week and a Kt/V value ≥1.2 for patients undergoing hemodialysis three times a week, (4) no malnutrition, as assessed by the Malnutrition Inflammation Score (MIS), (5) no history of parathyroidectomy, (6) proficiency in Thai and the ability to use the Line application independently or with assistance to facilitate communication and understand the study procedures, and (7) willingness to participate in the study. The exclusion criteria were: (1) inability to participate in activities due to a planned change of address, change in hemodialysis center, or hospital admission.

### 2.3 Sample size

The sample size was calculated for an RCT with continuous outcome variables (39, 40). A minimum sample size of 34 was required to achieve 80% power, with a type-I error (α) of 0.05 and a two-sided significance level. Considering a dropout rate of 15%, the sample size was set at 40 participants per group. The effect size, representing the mean difference in serum phosphorus between the intervention and control groups, was −0.59. This value was extracted from the results of a pilot study conducted at a hemodialysis center in 2021, revealing that the usual care approach resulted in an average decrease of 0.41 mg/dL, with a standard deviation (SD) of 0.86, in serum phosphorus levels over 6 months.

### 2.4 Randomization and blinding

**Figure 1:**
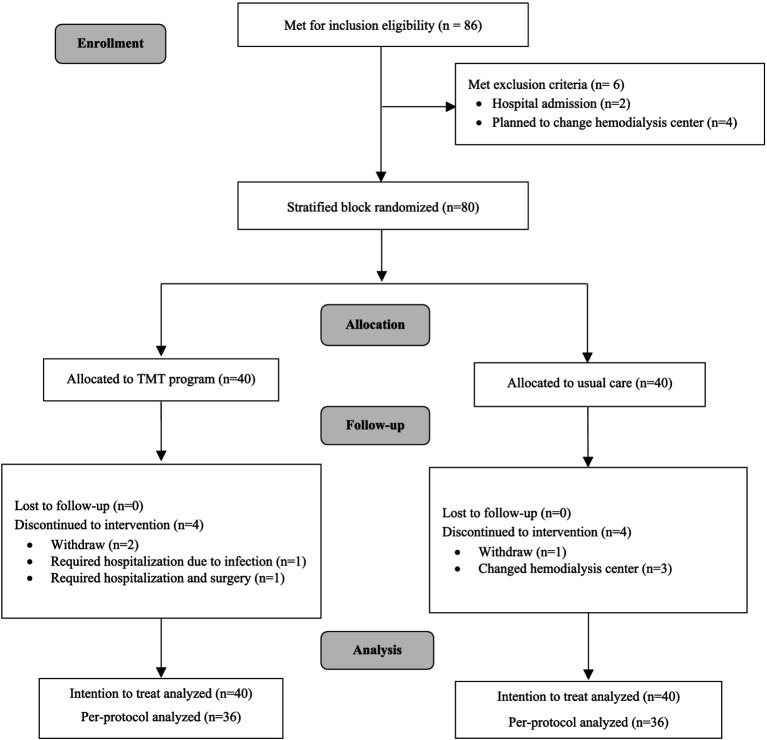
Flow diagram of randomization.

The enrollment process involved direct contact with 86 patients meeting the inclusion criteria, identified through brochure distribution to 244 patients across three hemodialysis centers. Six participants were excluded, two due to hospital admission and four who planned to change hemodialysis centers. After providing adequate information and ensuring that 80 participants had given informed consent, stratified block randomization was employed to balance the distribution of participants, dividing them based on the number of hemodialysis sessions per week (2 times and 3 times). The website sealedenvelope.com was employed to block randomize the sample using block sizes of 2 and 4 for a 2-arm parallel intervention trial (41). To conceal allocation, participants were assigned code numbers. The investigator then selected codes from the generated allocation sequences without knowing the participants’ identities, and partially blinded was used with outcome assessors being blinded to the participant’s group assignment. The 80 participants were randomly assigned at a 1:1 ratio, with 40 in the intervention group and 40 in the control group (Figure 1).

### 2.5 Procedure and intervention development

Before the study commenced, participants were assessed for readiness to change (42, 43), and both primary and secondary outcomes were measured at baseline, during the 12-week progress monitoring, and at the study endpoint at 24 weeks.

The TMT program was designed for the intervention group by integrating insights from the literature and expert interviews. This program combines the transtheoretical model and motivational interviewing to facilitate participants’ behavioral changes in dietary phosphorus restriction and adherence to phosphate binders to reduce serum phosphorus levels. Previous studies focused on behavior change and improving outcomes (27, 29, 31–33) were reviewed to comprehend hyperphosphatemia and its relevance. Subsequently, interviews with nephrologists and hemodialysis nurses from three different centers were carried out to delve into the complexities of hyperphosphatemia and discuss behavioral aspects associated with ESRD patients undergoing hemodialysis.

In the context of the COVID-19 pandemic, recognizing the need to prevent the spread of the virus, the program explored the integration of telehealth methodologies and their potential to enhance patient engagement and improve outcomes (35, 37, 44), using both group and individual video conferences, video clips, and text messages. Based on patient preferences for communication tools, the LINE application (45), was considered due to its user-friendly features and common use in Thailand (46).

Expert consultations with psychology, nephrology, and hemodialysis nurses were sought to refine the program’s design for validity and feasibility. Additionally, the researcher underwent training in motivational interviewing in healthcare, incorporating best practices into the program’s development and implementation.

#### 2.5.1 Intervention group (TMT program)

**Figure 2:**
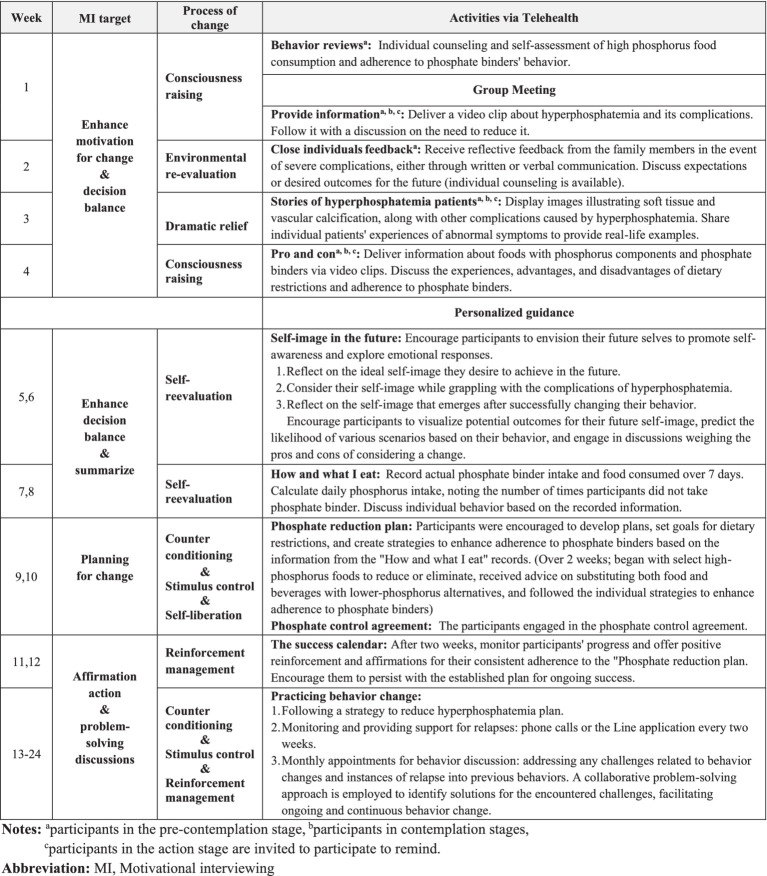
Schematic overview of the TMT program for the intervention group.

Figure 2 provides a comprehensive overview of the 24-week TMT program, incorporating activities from the process of change (47–50) aligned with participants’ readiness to change and the goals of motivational interviewing.

In the initial 4 weeks, participants engaged in 20-to-30-min weekly group discussions via video conferences. Group sizes ranged from 2 to 5 participants, accommodating the convenience of the participants each week; flexible scheduling ensured that all participants engaged and participated in all group discussion activities. Before these sessions, participants received and viewed three video clips. The researcher developed these clips and reviewed them with experts, including two nephrologists, a hemodialysis nurse, and a dietitian. The first clip provided a comprehensive explanation of the causes, symptoms, and complications of hyperphosphatemia and initiated a discussion during the first week on the importance of reducing serum phosphorus levels. The second clip emphasized the importance of dosing phosphate binders with meals for effectiveness, and the third illustrated varying phosphate levels in different foods and provided guidance on distinguishing absorbable phosphate. This included differentiating between organic phosphate found in the proteins of plants and animals and inorganic phosphorus, a chemical compound added to food during processing (51). Participants discussed their experiences and the advantages and disadvantages of adhering to phosphate binders and dietary restrictions. They also viewed images illustrating soft tissue and vascular calcification, along with other complications caused by hyperphosphatemia, and shared individual patients’ experiences of abnormal symptoms. These activities aimed to enhance participants’ understanding of hyperphosphatemia, provide a clear and well-informed perspective on their condition, and motivate positive changes. Details of the group discussions are outlined in Figure 2.

From the 5^th^ to the 24^th^ week, participants received personalized guidance to explore their desires, reasons, abilities, and needs for change. Knowledge exchange facilitated decision-making discussions about behavior change. Participants were encouraged to create individualized plans tailored to their lifestyles for behavior change. Patient progress was monitored over the 24 weeks, and any challenges or issues were discussed and addressed collaboratively. Details of personalized guidance are outlined in Figure 2.

Motivational interviewing techniques were consistently employed in communication with participants throughout all activities to foster collaboration and enhance decision balance, motivation, and goal orientation. This entailed using active listening, open-ended questions, empathy expression, affirmation, and summarization, all of which encouraged patients to express change talk and implementation (31–33).

#### 2.5.2 Control group (usual care)

Participants in the control group received usual care and personalized information designed to enhance their understanding of hyperphosphatemia and its potential complications. This information emphasized the importance of adhering to phosphate binders with meals for effective management. All interactions were conducted using personal protective equipment following COVID-19 outbreak prevention guidelines. Additionally, participants were given recommendations for alternative food choices to reduce phosphate intake and guidance on varying phosphate levels in different foods. Visual pamphlets illustrating phosphate content, ranging from low to high, were also provided. The researcher distributed printed educational materials to participants in the control group, containing the same content and images as the three video clips used in the experimental group. Participants in the control group could review the information independently after receiving explanations from the staff. Following the 12^th^ week of serum phosphorus level monitoring, an additional session was conducted to offer further clarification and individualized support to each participant based on serum phosphorus levels.

### 2.6 Outcomes measurement

The primary outcome was serum phosphorus levels obtained from blood tests. Secondary outcomes included knowledge of hyperphosphatemia management, dietary consumption behavior, self-efficacy regarding phosphate binder adherence, phosphate binder adherence, and the level of readiness to change. These outcomes were assessed using questionnaires administered by assessors at baseline, monitored at 12 weeks, and evaluated at the 24-week endpoint. The reliability of all questionnaires was established before their use in the study through a pilot study conducted with ESRD patients undergoing hemodialysis. The knowledge and dietary consumption behavior questionnaires, which were newly developed, underwent content validation and received approval from two nephrologists and an expert nephrology nurse specializing in hemodialysis. The scores from these questionnaires were calculated as percentages for statistical analysis.

Knowledge of hyperphosphatemia management was assessed using a questionnaire consisting of two parts covering key factors related to hyperphosphatemia management, including diseases, drugs, diet, and dialysis (16, 52, 53). Part one encompassed 15 multiple-choice questions that evaluated participants’ comprehension of hyperphosphatemia. Part two consisted of 25 items, focusing on the knowledge of high-phosphate foods and beverages in Thailand. The questionnaire demonstrated content validity, achieving an index of item-objective congruence (IOC) value of 0.91. The internal consistency reliability was measured using the Kuder–Richardson Formula 20 (KR-20), resulting in a value of 0.73 for part one and 0.71 for part two.

Dietary consumption behavior was assessed using food frequency questionnaires (FFQ) (54) tailored for Thai food, which aimed to evaluate participants’ dietary habits concerning foods containing phosphorus. The FFQ comprises 30 questions measured on a 7-point Likert scale, with a higher score indicating better dietary practices. Content validation demonstrated an IOC value of 0.90 and a Cronbach’s alpha reliability of 0.74. The phosphorus food frequency questionnaire (P-FFQ) assessed the levels of organic and inorganic phosphate from food and beverages, based on the booklet for phosphorus content for CKD and hemodialysis patients in Thailand from the Thai Dietetic Association (55, 56).

The self-efficacy of phosphate binder adherence was assessed using the Self-Efficacy for Appropriate Medication Use Scale (SEAMS), originally developed in 2007 (57) and translated into Thai in 2014 (58). The Cronbach’s alpha reliability was 0.89 in the original study and 0.91 in the pilot study with ESRD patients undergoing hemodialysis. The instrument consists of 13 questions assessed on a 3-point Likert scale, and the scores are categorized into three levels: low, moderate, and high confidence.

The questionnaires for phosphate binder adherence and the level of readiness to change were adjusted and back-translated into Thai by an English expert. Their content validity was approved by two nephrologists, a clinical psychiatrist, and a nephrology nurse specializing in hemodialysis care.

Adherence to phosphate binders was assessed using the Simplified Medication Adherence Questionnaire (SMAQ) (59), which was previously employed to evaluate phosphate binder adherence in CKD patients undergoing hemodialysis in 2010 (60). The SMAQ comprises six questions with a Cronbach’s alpha reliability of 0.71. A total score below 6 indicates nonadherence.

The level of readiness to change was assessed using a 12-item questionnaire adapted from the readiness-to-change questionnaire (treatment version) (42, 43). All items were measured on a 5-point Likert scale, with a Cronbach’s alpha reliability of 0.78. The questions were based on the transtheoretical model, and participants were categorized into three stages: pre-contemplation, contemplation, and action, based on their readiness to reduce or eliminate phosphorus-containing foods from their diets. Participants who achieved the same score in each stage were classified into more advanced stages.

### 2.7 Statistical analysis

The baseline demographic and clinical characteristics were analyzed using descriptive statistics. Mean differences in serum phosphorus levels, knowledge of hyperphosphatemia management, and dietary consumption behavior scores for foods containing phosphorus between the TMT program and usual care groups were evaluated using linear regression unadjusted and adjusted analyses for age and gender. Fisher’s exact test was employed to assess differences in phosphate binder adherence, self-efficacy in the appropriate use of phosphate binders, and readiness for change. An intention-to-treat (ITT) analysis was performed, utilizing the first baseline value observation for comparison. Stata 18.0 software (StataCorp, 2023, Stata Statistical Software: Release 18, College Station, TX: StataCorp LLC) was used for all data analysis.

## 3 Result

### 3.1 Population and loss to follow-up

This trial, conducted from March to December 2022, included 80 patients, with an equal number (n = 40) randomly allocated to the TMT program and the usual care group. At the study endpoint (24 weeks), 80 participants were included in the intention-to-treat (ITT) analysis, while the per-protocol (PP) analysis was conducted with 72 participants (90% of all participants). In the TMT program group, there were two dropouts, and 2 participants had prolonged hospital admissions, rendering them unable to participate in all activities. In the usual care group, 1 participant dropped out, and 3 participants had to move out during the study. All dropouts occurred after 3 months due to individual inconveniences related to work, affecting their ability to participate in the activities.

### 3.2 Participant outcomes

The baseline sociodemographic characteristics presented in Table 1 revealed similarities with slight differences between the two groups. The TMT program group had a slightly higher number of patients under 50 years old and a higher proportion of male participants. However, clinical characteristics in Table 2 demonstrated similarities between the two groups.

**Table 1:** Sociodemographic characteristics of the participants (*n* = 80).

Characteristics	TMT program (*n* = 40)	Usual care (*n* = 40)
	*n* (%)	*n* (%)
Age group (year)		
<50	21 (52.5)	14 (35.0)
50–59	5 (12.5)	12 (30.0)
≥60	14 (35.0)	14 (35.0)
Male gender	27 (67.5)	17 (42.5)
Hemodialysis center		
Site 1	21 (52.50)	21 (52.50)
Site 2	9 (22.50)	8 (20.00)
Site 3	10 (25.00)	11 (27.50)
Hemodialysis per week (4 h/time)		
2 times/week	15 (37.5)	15 (37.5)
3 times/week	25 (62.5)	25 (62.5)
Level of education		
Uneducated	0	1 (2.5)
Primary school	19 (47.5)	21 (52.5)
Junior high school	5 (12.5)	5 (12.5)
Senior high school	5 (12.5)	6 (15.0)
Higher education (College or University)	11 (27.5)	7 (17.5)
Marital status		
Unmarried	11 (27.5)	15 (37.5)
Married	29 (72.5)	25 (62.5)
Occupation		
Unemployed	13 (32.5)	16 (40.0)
Employee	27 (67.5)	24 (60.0)
Sufficient income (self-report)	24 (60.0)	22 (55.0)
Caregiver	16 (40.0)	11 (27.5)
Social support (self-report)	40 (100.0)	39 (97.5)
Smoke		
Non-smoker	31 (77.5)	33 (82.5)
Previous smoker	9 (22.5)	7 (17.5)
Current smoker	0	0
Alcohol		
No	37 (92.5)	36 (90.0)
Yes	3 (7.5)	4 (10.0)

**Table 2:** Clinical characteristics of the participants (*n* = 80).

Characteristics	TMT program (*n* = 40)	Usual care (*n* = 40)
	*n* (%)	*n* (%)
Dialysis vintage (year), mean (SD)	4.67 (3.89)	6.12 (5.32)
Kt/V, mean (SD)	1.87 (0.38)	1.93 (0.31)
eGFR (ml/min/1.73 m^2^), mean (SD)	4.39 (1.48)	3.98 (1.18)
Urine output (ml/day)		
0	14 (35.0)	18 (45.0)
≤100	12 (30.0)	13 (32.5)
>100	14 (35.0)	9 (22.5)
BMI (kg/m^2^), mean (SD)	23.32 (3.91)	23.78 (4.99)
nPCR (g/kg/day), mean (SD)	1.27 (0.26)	1.32 (0.28)
TIBC (mg/dl), mean (SD)	226.25 (33.07)	228.35 (37.41)
Serum albumin (g/dl), mean (SD)	4.16 (0.31)	4.15 (0.31)
Serum calcium (mg/dl), mean (SD)	8.68 (1.22)	8.93 (0.93)
iPTH (pg/ml), median (IQR)	614.35 (386.55, 1,185.65)	560.65 (331.45, 770.45)
Received Vitamin D (Oral used)	19 (47.5)	21 (52.5)
Comorbidities		
Diabetes mellitus	16 (40.0)	15 (37.5)
Hypertension	29 (72.5)	31 (77.5)
Dyslipidemia	12 (30.0)	9 (22.5)
Heart disease (coronary or structural diseases)	6 (15.0)	5 (12.5)
Personal history of disease of CVA	5 (12.5)	2 (5.0)
The total number of drugs taken/meal without phosphate binders (tablet), mean (SD)	7.73 (3.99)	7.80 (2.92)
The number of phosphate-binding pills taken/dose (tablet), mean (SD)	1.48 (0.88)	1.39 (0.75)
Phosphate binders		
Calcium carbonate	30 (75.0)	30 (75.0)
Aluminium hydroxide	13 (32.5)	11 (27.5)
Sevelamer carbonate	3 (7.5)	2 (5.0)
Dosage of phosphate binders/day (mg), mean (SD)		
Calcium carbonate	2,850.00 (1,231.06)	2,979.17 (1,925.35)
Aluminium hydroxide	2,538.46 (1,282.28)	2,318.20 (1,031.33)
Sevelamer carbonate	2,400.00 (0.00)	2,400.00 (0.00)
The usage of phosphate binders		
No	2 (5.0)	2 (5.0)
Using 1 phosphate binder	30 (75.0)	33 (82.5)
Using ≥2 phosphate binders	8 (20.0)	5 (12.5)

Continuous data are presented as mean (SD) or median (interquartile range), as appropriate. Categorical data are presented as numbers (%). Kt/V, Volume of plasma cleared of urea divided by the urea distribution volume; eGFR, estimated glomerular filtration rate;BMI, Body Mass Index; iPTH, intact Parathyroid hormone; nPCR, Normalized Protein Catabolic Rate; TIBC, Total iron binding capacity; CVA, cerebrovascular accident.

**Figure 3:**
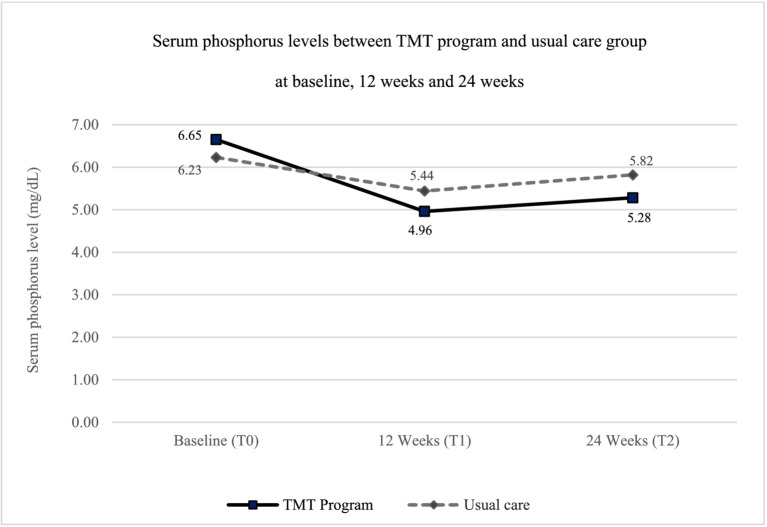
Graph of serum phosphorus levels for the TMT program and usual care groups.

The study assessed outcomes at the 12-week follow-up (∆T1–T0) and the 24-week endpoint (∆T2–T0). The decrease in serum phosphorus levels in the TMT program group was greater than in the usual care group at both time points compared to baseline (Figure 3). The mean difference in serum phosphorus levels between the TMT program and the usual care group was statistically significant, with mean differences of −0.84 (95% CI: −1.61, −0.07) at the 12-week follow-up and −1.03 (95% CI: −1.77, −0.29) at the 24-week endpoint, after adjusting for gender and age (Table 3).

**Table 3:** Comparison of serum phosphorus levels, knowledge about hyperphosphatemia management, and dietary consumption behavior of foods containing phosphorus between two groups (*n* = 80).

Outcomes	TMT program Mean (SD)	Usual care Mean (SD)	Mean difference between groups	(95% CI)	Adjusted mean difference between groups^a^	(95% CI)
Level of serum phosphorus (mg/dL)						
Baseline (T0)	6.65 (1.49)	6.23 (1.31)	-	-	-	-
At 12^th^ week (T1)	4.96 (1.53)	5.44 (1.39)	-	-	-	-
At 24^th^ week (T2)	5.28 (1.61)	5.82 (1.48)	-	-	-	-
∆T1–T0	−1.69 (1.41)	−0.79 (1.86)	−0.90	(−1.64, −0.16)	−0.84	(−1.61, −0.07)
∆T2–T0	−1.37 (1.53)	−0.41 (1.66)	−0.97	(−1.68, −0.25)	−1.03	(−1.77, −0.29)
The score of knowledge about hyperphosphatemia management						
Baseline (T0)	58.45 (18.71)	55.64 (17.07)	-	-	-	-
At 12^th^ week (T1)	78.22 (11.48)	67.59 (12.67)	-	-	-	-
At 24^th^ week (T2)	81.31 (10.49)	72.27 (14.58)	-	-	-	-
∆T1–T0	19.77 (14.56)	11.95 (10.05)	7.82	(2.25, 13.39)	7.46	(2.10, 12.82)
∆T2–T0	22.86 (19.13)	16.63 (17.54)	6.23	(−1.94, 14.39)	7.02	(−1.03, 15.07)
The score of dietary consumption behavior of foods containing phosphorus						
Baseline (T0)	58.44 (7.84)	63.38 (10.70)	-	-	-	-
At 12^th^ week (T1)	76.00 (7.34)	67.19 (11.77)	-	-	-	-
At 24^th^ week (T2)	76.35 (8.79)	67.80 (11.43)	-	-	-	-
∆T1–T0	17.56 (9.21)	3.81 (12.42)	13.75	(8.88, 18.62)	13.33	(8.28, 18.37)
∆T2–T0	17.91 (11.03)	4.42 (11.26)	13.49	(8.53, 18.45)	13.48	(8.41, 18.57)

a Adjusted for age and gender.

Knowledge of hyperphosphatemia management did not differ significantly between the TMT program group and the usual care group at the 24-week endpoint, with a mean difference of 7.02 (95% CI: −1.03, 15.07). However, dietary consumption behavior related to phosphorus-containing foods consistently improved in the TMT program group at both 12 and 24 weeks, showing a significant increase compared to the usual care group. The mean differences were 13.33 (95% CI: 8.28, 18.37) at 12 weeks and 13.48 (95% CI: 8.41, 18.57) at 24 weeks, after adjusting for gender and age (Table 3).

Table 4 presents significant differences between both groups at the 24-week endpoint in each outcome: readiness to change (90.0% vs. 47.5%, *p*-value <0.001), self-efficacy in the appropriate use of phosphate binders (81.6% vs. 55.3%, *p*-value = 0.025), and adherence to phosphate binders (73.7% vs. 34.2%, *p*-value = 0.001).

**Table 4:** Comparison of the levels of readiness to change, self-efficacy for the appropriate use of phosphate binders, and adherence to phosphate binders between two groups.

Outcomes	Baseline n (%)		*p*-value^a^	Monitor 12 weeks *n* (%)		*p*-value^a^	Endpoint 24 weeks *n* (%)		*p*-value^a^
	TMT program	Usual care		TMT program	Usual care		TMT program	Usual care	
The level of readiness to change (*n* = 80)									
Precontemplation	3 (7.5)	3 (7.5)	0.939	0	0	0.222	0	1 (2.5)	< 0.001
Contemplation	15 (37.5)	13 (32.5)		9 (22.5)	15 (37.5)		4 (10.0)	20 (50.0)	
Action	22 (55.0)	24 (60.0)		31 (77.5)	25 (62.5)		36 (90.0)	19 (47.5)	
The level of self-efficacy for appropriate use of phosphate binders (*n* = 76)									
Low	2 (5.2)	1 (2.6)	> 0.999	0	1 (2.6)	0.061	0	0	0.025
Moderate	18 (47.4)	18 (47.4)		11 (28.9)	19 (50.0)		7 (18.4)	17 (44.7)	
High	18 (47.4)	19 (50.0)		27 (71.1)	18 (47.4)		31 (81.6)	21 (55.3)	
Adherence to phosphate binders (*n* = 76)									
Non-adherence	29 (76.3)	27 (71.1)	0.795	13 (34.2)	27 (71.1)	0.003	10 (26.3)	25 (65.8)	0.001
Adherence	9 (23.7)	11 (28.9)		25 (65.8)	11 (28.9)		28 (73.7)	13 (34.2)	

a *p-values from Fisher’s exact test.*

The results of the PP analysis of the primary and secondary outcomes were consistent with the results of the ITT analysis. No adverse events related to the intervention, including malnutrition or hypophosphatemia, were observed in the present study.

## 4 Discussion

The study results suggest that the TMT program significantly reduced serum phosphorus levels, improved dietary phosphorus consumption behavior, increased self-efficacy for phosphate binder use, and enhanced phosphate binder adherence among ESRD patients undergoing chronic hemodialysis. Moreover, the study is among the first in Asia to initiate research focused on decreasing serum phosphorus levels through telehealth during the COVID-19 pandemic.

### 4.1 The effect of the intervention on serum phosphorus levels

Increased knowledge about a low-phosphate diet and proper phosphate binder intake may not be sufficient for managing serum phosphorus levels (20). Therefore, this study explored integrating the transtheoretical model with motivational interviewing to help participants change these behaviors and reduce serum phosphorus levels. The transtheoretical model comprises five stages of change and acknowledges an individual’s readiness to change (26, 61) before offering guidance through tailored activities that align with participants’ stages to promote behavioral change. This method extends beyond the mere provision of knowledge.

In the precontemplation stage, activities must address insufficient knowledge, recognize health issues, provide motivation, and reduce the underestimation of behavior change. The contemplation stage utilizes activities to resolve ambivalence and enhance decision balance, fostering increased recognition and self-reevaluation. These activities then transition into the preparation stage, where participants are offered willpower and choices, allowing them to implement their plans. During the action stage, participants follow their planned activities to help them adhere to their plans for as long as possible, facilitating the shift to maintenance and reducing relapse (47–50). These activities, integral to the processes of change, serve as a bridge, aiding patients in maintaining action and behavioral changes (50). They enhance individuals’ progress in reducing serum phosphorus levels, which is consistent with findings from other studies (27–29).

The study also integrated motivational interviewing to establish effective communication and cultivate a supportive, empathetic, and non-judgmental atmosphere, aiming to decrease patients’ suffering, diminish negative emotions, and prevent resistance (11, 18, 30, 62). A guiding style of motivational interviewing encourages patients’ autonomy in choosing their path while offering support, information, and reasons for change, encompassing desires, wishes, hopes, needs, and the importance of change. The motivational interviewing technique involves active listening and open-ended questions to help promote increased focus and self-exploration of participants’ concerns. Expressions of empathy, affirmation, and praise are incorporated to recognize participants’ efforts and demonstrate a profound understanding of their challenges. Summarization facilitates reflection and clarification of goals (63). The researcher serves as a guide, allowing patients to choose the direction of their journey while providing support and autonomy, enhancing flexibility and reducing resistance to promoting their self-efficacy and behavior change (30, 64, 65). The results of the activities helped participants understand and recognize the risks associated with complications from hyperphosphatemia while also fostering awareness of their desires and life goals. This approach generated motivation and enabled participants to decide to change their behavior.

Our findings revealed that the TMT program group consistently achieved higher adherence and progression to the action stage, maintaining this trend over a more extended period than the usual care group at both the 12-week and 24-week time points. These results show that the participants successfully made positive changes in their lives as they progressed through stages of change, consistent with findings from studies of other diseases (26). In particular, the TMT program group consistently demonstrated improvements in dietary consumption of phosphorus-containing foods, self-efficacy for the appropriate use of phosphate binders, and high adherence rates, leading to a more significant reduction in serum phosphorus levels than with health education alone. This approach reduces the likelihood of relapse or reverting to old behaviors, as evidenced by the decrease in serum phosphorus levels in the TMT group compared to the control group. It is essential to recognize that efforts to change participants’ behavior are not a one-time endeavor and may encounter obstacles that can lead to frustration and reduced sustained effort. While helping patients decide to change their behavior is challenging, maintaining these changes is even more difficult. Healthcare professionals should avoid judging patients and strive to understand their issues beyond basic self-care knowledge. Patients need encouragement, empathy and respect (11) to build their confidence and promote lasting behavioral changes over time.

Although all activities were conducted via telehealth, the outcomes were similar to a study on virtual education during the COVID-19 pandemic for cardiac rehabilitation patients who reported a significant increase in changed behavior. Both interventions showed the potential to improve self-efficacy, tailor activities (66), and provide flexibility in time and location (67). Moreover, although participants in the TMT program were slightly younger and had more male participants than the control group, these factors can significantly influence non-adherence to treatment plans and dietary restrictions among hemodialysis patients (68–71). However, after adjusting for age and gender, the TMT program group exhibited a reduction in serum phosphorus levels compared to the control group. This observation suggests that the TMT program may be more effective in promoting engagement in activities than the usual care approach. Telehealth communication can help alleviate limitations related to time and location for appointments, which is particularly beneficial for younger patients. These patients typically have better access to technology and face time constraints due to their careers. As a result, participating in activities becomes more accessible, leading to better adherence to treatment plans and improved clinical outcomes.

### 4.2 Strength

This study had several strengths. Participants were recruited from multiple hemodialysis centers in various geographic areas. The study employed a multimodal approach, integrating the transtheoretical model, motivational interviewing, and telehealth for communication through the Line application during the COVID-19 pandemic. This period presented challenges, including limited access to food choices and increased consumption of processed foods-one of the causes of hyperphosphatemia, while traditional in-person interactions were limited. However, the TMT program proved effective in reducing serum phosphorus.

The flexible approach allowed participants to access video conferences and review the video content at their convenience, ensuring a more effective and convenient learning experience. Participants could share these video clips with their caregivers and family members, fostering family support. This not only enhanced the learning experience but also minimized the risk of viral infection. Since assessors were blind to study conditions, bias in assessing the study outcomes was reduced.

### 4.3 Limitations

This study had several limitations. First, providing educational video clips via telehealth without blinding participants may introduce contamination bias. This could affect the differentiation in knowledge about hyperphosphatemia management between the two groups and dilute the effect of the TMT program due to the potential sharing of video clips. Second, while the stage of change was assessed using the readiness-to-change questionnaire (42, 43), it could not specify the duration participants spent in each stage. Follow-ups were conducted at 12 weeks and at the endpoint to evaluate progress and participants’ stages. Additionally, using questionnaires to assess participants’ behavior may introduce recall bias; this was minimized by incorporating food image-based prompts to help remind participants. Lastly, although this study’s findings provide meaningful insights for many hemodialysis patients, those who cannot utilize telehealth services may not have access to them.

### 4.4 Recommendations

Telehealth is crucial in pandemic situations as it helps bridge gaps in healthcare. The current study lasted 24 weeks; therefore, future research should assess the sustainability of the TMT program aimed at controlling serum phosphorus levels. Exploring other communication methods, such as text messages or applications, would be an interesting avenue for further studies.

### 4.5 Conclusion

In this RCT, the TMT program demonstrated a reduction in serum phosphorus levels compared to usual care, offering an effective strategy for managing hyperphosphatemia in ESRD patients undergoing hemodialysis during the COVID-19 pandemic. Additionally, the program improved dietary consumption of phosphorus-containing foods, increased self-efficacy, and enhanced adherence rates to phosphate binders, positively impacting stage-of-change behavior.

## Data Availability

The datasets presented in this article are not readily available because the data analyzed in this study is subject to the following licenses/restrictions: Access to the data must be approved by the Institutional Review Board of the Faculty of Medicine, Chulalongkorn University. Requests to access the datasets should be directed to Vitool Lohsoonthorn, vitool.l@chula.ac.th.

## References

[1] Levey AS Coresh J. Chronic kidney disease. Lancet. (2012) 379:165–80. doi: 10.1016/S0140-6736(11)60178-5, PMID: 21840587

[2] Thomas R Kanso A Sedor JR. Chronic kidney disease and its complications. Prim Care. (2008) 35:329–44. doi: 10.1016/j.pop.2008.01.008, PMID: 18486718 PMC2474786

[3] Kidney Disease: Improving Global Outcomes (KDIGO) CKD-MBD Update Work Group. KDIGO. Clinical practice guideline update for the diagnosis, evaluation, prevention, and treatment of chronic kidney disease-mineral and bone disorder (CKD-MBD). Kidney Int Suppl. (2017) 7:1–59. doi: 10.1016/j.kisu.2017.04.001, PMID: 30675420 PMC6340919

[4] Cozzolino M Ciceri P Galassi A. Hyperphosphatemia: a novel risk factor for mortality in chronic kidney disease. Ann Transl Med. (2019) 7:55. doi: 10.21037/atm.2018.06.50, PMID: 30906759 PMC6389588

[5] Rodriguez-Benot A Martin-Malo A Alvarez-Lara MA Rodriguez M Aljama P. Mild hyperphosphatemia and mortality in hemodialysis patients. Am J Kidney Dis. (2005) 46:68–77. doi: 10.1053/j.ajkd.2005.04.006, PMID: 15983959

[6] Rastogi A Bhatt N Rossetti S Beto J. Management of hyperphosphatemia in end-stage renal disease: a new paradigm. J Ren Nutr. (2021) 31:21–34. doi: 10.1053/j.jrn.2020.02.003, PMID: 32386937

[7] Hiyamuta H Yamada S Taniguchi M Tokumoto M Tsuruya K Nakano T. Association of hyperphosphatemia with an increased risk of sudden death in patients on hemodialysis: ten-year outcomes of the Q-cohort study. Atherosclerosis. (2021) 316:25–31. doi: 10.1016/j.atherosclerosis.2020.11.020, PMID: 33260008

[8] Berndt TJ Schiavi S Kumar R. Phosphatonins and the regulation of phosphorus homeostasis. Am J Physiol Ren Physiol. (2005) 289:F1170–82. doi: 10.1152/ajprenal.00072.2005, PMID: 16275744

[9] Shaman AM Kowalski SR. Hyperphosphatemia Management in Patients with chronic kidney disease. Saudi Pharm J. (2016) 24:494–505. doi: 10.1016/j.jsps.2015.01.009, PMID: 27330380 PMC4908098

[10] Vervloet MG van Ballegooijen AJ. Prevention and treatment of hyperphosphatemia in chronic kidney disease. Kidney Int. (2018) 93:1060–72. doi: 10.1016/j.kint.2017.11.036, PMID: 29580635

[11] Umeukeje EM Mixon AS Cavanaugh KL. Phosphate-control adherence in hemodialysis patients: current perspectives. Patient Prefer Adheren. (2018) 12:1175–91. doi: 10.2147/PPA.S145648, PMID: 30013329 PMC6039061

[12] Copland M Komenda P Weinhandl ED McCullough PA Morfin JA. Intensive hemodialysis, mineral and bone disorder, and phosphate binder use. Am J Kidney Dis. (2016) 68:S24–32. doi: 10.1053/j.ajkd.2016.05.024, PMID: 27772640

[13] The National Kidney Foundation Kidney Disease Outcomes Quality Initiative (2003). KDOQI clinical practice guidelines for bone metabolism and disease in chronic kidney disease 2003. Available at: http://kidneyfoundation.cachefly.net/professionals/KDOQI/guidelines_bone/guide3.htm (Accessed July 29, 2021). 10.1053/j.ajkd.2006.11.027 17261428

[14] Karavetian M de Vries N Rizk R Elzein H. Dietary educational interventions for management of hyperphosphatemia in hemodialysis patients: a systematic review and meta-analysis. Nutr Rev. (2014) 72:471–82. doi: 10.1111/nure.12115, PMID: 24920494

[15] Cupisti A Ferretti V D'Alessandro C Petrone I Di Giorgio A Meola M. Nutritional knowledge in hemodialysis patients and nurses: focus on phosphorus. J Ren Nutr. (2012) 22:541–6. doi: 10.1053/j.jrn.2011.11.003, PMID: 22296916

[16] Toussaint ND Pedagogos E Beavis J Becker GJ Polkinghorne KR Kerr PG. Improving CKD-MBD management in haemodialysis patients: barrier analysis for implementing better practice. Nephrol Dial Transplant. (2011) 26:1319–26. doi: 10.1093/ndt/gfq602, PMID: 20935019

[17] Collinson A McMullan M Tse WY Sadler H. Managing serum phosphate in haemodialysis patients: time for an innovative approach? Eur J Clin Nutr. (2014) 68:392–6. doi: 10.1038/ejcn.2013.283, PMID: 24424075

[18] Kalantar-Zadeh K. Patient education for phosphorus management in chronic kidney disease. Patient Prefer Adheren. (2013) 7:379–90. doi: 10.2147/PPA.S43486, PMID: 23667310 PMC3650565

[19] Ghimire S Castelino RL Lioufas NM Peterson GM Zaidi ST. Nonadherence to medication therapy in haemodialysis patients: a systematic review. PLoS One. (2015) 10:e0144119. doi: 10.1371/journal.pone.0144119, PMID: 26636968 PMC4670103

[20] Lim E Hyun S Lee JM Kim S Lee MJ Lee SM. Effects of education on low-phosphate diet and phosphate binder intake to control serum phosphate among maintenance hemodialysis patients: a randomized controlled trial. Kidney Res Clin Pract. (2018) 37:69–76. doi: 10.23876/j.krcp.2018.37.1.69, PMID: 29629279 PMC5875578

[21] Davison SN Jhangri GS. Existential and supportive are needs among patients with chronic kidney disease. J Pain Symptom Manag. (2010) 40:838–43. doi: 10.1016/j.jpainsymman.2010.03.015, PMID: 20739142

[22] Fradelos EC Tzavella F Koukia E Papathanasiou IV Alikari V Stathoulis J. Integrating chronic kidney disease patient's spirituality in thier care: health benefits and research perspectives. Mater Soc. (2015) 27:354–8. doi: 10.5455/msm.2015.27.354-358, PMID: 26622206 PMC4639341

[23] Prochaska JO Velicer WF Fava JL Rossi JS Tsoh JY. Evaluating a population-based recruitment approach and a stage-based expert system intervention for smoking cessation. Addict Behav. (2001) 26:583–602. doi: 10.1016/S0306-4603(00)00151-9, PMID: 11456079

[24] Baumann S Gaertner B Schnuerer I Bischof G John U Freyer-Adam J. How well do TTM measures work among a sample of individuals with unhealthy alcohol use that is characterized by low readiness to change? Psychol Addict Behav. (2013) 27:573–83. doi: 10.1037/a0029368, PMID: 22867296

[25] Johnson SS Paiva AL Cummins CO Johnson JL Dyment SJ Wright JA. Transtheoretical model-based multiple behavior intervention for weight management: effectiveness on a population basis. Prev Med. (2008) 46:238–46. doi: 10.1016/j.ypmed.2007.09.010, PMID: 18055007 PMC2327253

[26] Li X Yang S Wang Y Yang B Zhang J. Effects of a transtheoretical model - based intervention and motivational interviewing on the management of depression in hospitalized patients with coronary heart disease: a randomized controlled trial. BMC Public Health. (2020) 20:420. doi: 10.1186/s12889-020-08568-x, PMID: 32228532 PMC7106625

[27] Karavetian M de Vries N Elzein H Rizk R Bechwaty F. Effect of behavioral stage-based nutrition education on management of osteodystrophy among hemodialysis patients, Lebanon. Patient Educ Couns. (2015) 98:1116–22. doi: 10.1016/j.pec.2015.05.005, PMID: 26070468

[28] Rizk R Karavetian M Hiligsmann M Evers S. Effect of stage-based education provided by dedicated dietitians on hyperphosphataemeic haemodialysis patients: results from the nutrition education for management of osteodystrophy randomised controlled trial. J Hum Nutr Diet. (2017) 30:554–62. doi: 10.1111/jhn.12472, PMID: 28322468

[29] de Melo V Ribeiro P Miranda Hermsdorff HH Balbino KP de Paula Santos Epifânio A de Paula Jorge M. Effect of a nutritional intervention, based on transtheoretical model, on metabolic markers and food consumption of individuals undergoing hemodialysis. J Ren Nutr. (2020) 30:430–9. doi: 10.1053/j.jrn.2019.12.004, PMID: 32037084

[30] Rollnick S Miller WR Butler C. Motivational interviewing in health care: helping patients change behavior. New York: Guilford Publications (2008).

[31] Sanders KA Whited A Martino S. Motivational interviewing for patients with chronic kidney disease. Semin Dial. (2013) 26:175–9. doi: 10.1111/sdi.12052, PMID: 23406198 PMC3608718

[32] Barikani A Negarandeh R Moin M Fazlollahi MR. The impact of motivational interview on self-efficacy, beliefs about medicines and medication adherence among adolescents with asthma: a randomized controlled trial. J Pediatr Nurs. (2021) 60:116–22. doi: 10.1016/j.pedn.2021.04.020, PMID: 33932626

[33] García-Llana H Remor E del Peso G Celadilla O Selgas R. Motivational interviewing promotes adherence and improves wellbeing in pre-dialysis patients with advanced chronic kidney disease. J Clin Psychol Med Settings. (2014) 21:103–15. doi: 10.1007/s10880-013-9383-y, PMID: 24281770

[34] Li W-Y Chiu F-C Zeng J-K Li Y-W Huang S-H Yeh H-C. Mobile health app with social media to support self-management for patients with chronic kidney disease: prospective randomized controlled study. J Med Internet Res. (2020) 22:e19452. doi: 10.2196/19452, PMID: 33320101 PMC7772070

[35] Ong SW Jassal SV Miller JA Porter EC Cafazzo JA Seto E. Integrating a smartphone-based self-management system into usual care of advanced CKD. Clin J Am Soc Nephrol. (2016) 11:1054–62. doi: 10.2215/cjn.10681015, PMID: 27173169 PMC4891756

[36] Arora S Peters AL Agy C Menchine M. A mobile health intervention for inner city patients with poorly controlled diabetes: proof-of-concept of the TExT-MED program. Diabetes Technol Ther. (2012) 14:492–6. doi: 10.1089/dia.2011.0252, PMID: 22524591

[37] Tuot DS Boulware LE. Telehealth applications to enhance CKD knowledge and awareness among patients and providers. Adv Chronic Kidney Dis. (2017) 24:39–45. doi: 10.1053/j.ackd.2016.11.017, PMID: 28224941 PMC5324778

[38] Haapala I Barengo NC Biggs S Surakka L Manninen P. Weight loss by mobile phone: a 1-year effectiveness study. Public Health Nutr. (2009) 12:2382–91. doi: 10.1017/S1368980009005230 19323865

[39] Zhong B. How to calculate sample size in randomized controlled trial? J Thorac Dis. (2009) 1:51–4. PMID: 22263004 PMC3256489

[40] Wittes J. Sample size calculations for randomized controlled trials. Epidemiol Rev. (2002) 24:39–53. doi: 10.1093/epirev/24.1.39, PMID: 12119854

[41] Sealed Envelope Ltd (2022). Create a blocked randomisation list. Available at: https://www.sealedenvelope.com/simple-randomiser/v1/lists (Accessed April 4, 2022).

[42] Heather N Hönekopp J. A revised edition of the readiness to change questionnaire [treatment version]. Addict Res Theory. (2009) 16:421–33. doi: 10.1080/16066350801900321

[43] Richards DK Morera OF Cabriales JA Smith JC Field CA. Factor, concurrent and predictive validity of the readiness to change questionnaire [treatment version] among non-treatment-seeking individuals. Alcohol Alcohol. (2020) 55:409–15. doi: 10.1093/alcalc/agaa021, PMID: 32318693 PMC7307315

[44] Fakih El Khoury C Crutzen R Schols JMGA Halfens RJG Karavetian M. A dietary mobile app for patients undergoing hemodialysis: prospective pilot study to improve dietary intakes. J Med Internet Res. (2020) 22:e17817. doi: 10.2196/17817, PMID: 32706698 PMC7399958

[45] LINE Corporation (2021). LINE: calls & messages. Available at: https://play.google.com/store/apps/details?id=jp.naver.line.android&pli=1 (Accessed July 29, 2021).

[46] LINE Corporation (2018). Line q2 2018 earning results. Available at: https://scdn.line-apps.com/stf/linecorp/en/ir/all/FY18Q2_Presentation.EN.pdf (Accessed July 29, 2021).

[47] DiClemente CC Prochaska JO. Self-change and therapy change of smoking behavior: a comparison of processes of change in cessation and maintenance. Addict Behav. (1982) 7:133–42. doi: 10.1016/0306-4603(82)90038-7, PMID: 7102444

[48] Karl T. (2021). 10 processes of change-do you know what drives the 5 stages? Available at: https://r1learning.com/blog/2021/10-processes-of-change (Accessed December 22, 2022).

[49] DiClemente C. The transtheoretical model of intentional behaviour change. Drugs Alcohol Today. (2007) 7:29–33. doi: 10.1108/17459265200700007, PMID: 37227166

[50] Prochaska JO DiClemente CC. Stages and processes of self-change of smoking: toward an integrative model of change. J Consult Clin Psychol. (1983) 51:390–5. doi: 10.1037/0022-006X.51.3.390, PMID: 6863699

[51] Kalantar-Zadeh K Fouque D. Nutritional management of chronic kidney disease. N Engl J Med. (2017) 377:1765–76. doi: 10.1056/NEJMra1700312, PMID: 29091561

[52] Hjemås BJ Bøvre K Mathiesen L Lindstrøm JC Bjerknes K. Interventional study to improve adherence to phosphate binder treatment in dialysis patients. BMC Nephrol. (2019) 20:178. doi: 10.1186/s12882-019-1334-x, PMID: 31101020 PMC6525353

[53] Shi YX Si W Liu JD Gao M Wang SY Cheng M. Development and evaluation of the psychometric properties of the CKD-MBD knowledge and behavior (CKD-MBD-KB) questionnaire for patients with chronic kidney disease. J Pain Symptom Manag. (2016) 51:557–8.e2. doi: 10.1016/j.jpainsymman.2015.07.021, PMID: 26854994

[54] Cambridge Biomedical Research Centre (2021). Diet anthropometry and physical activity (DAPA) measurement toolkit, food frequency questionaires. Available at: https://www.measurement-toolkit.org/diet/subjective-methods/food-frequency-questionnaire# (Accessed July 29, 2021).

[55] Cherdrungsi Y. Development of phosphorus counting booklet for hemodialysis patient [master's thesis]. Nakhon Pathom: Mahidol University (2013).

[56] Cherdrungsi Y Pachotikarn C Taechangam S. (2021). Booklet for phosphorus content in food: Thai dietetic association. Available at: https://anyflip.com/qcpub/vurp/basic/ (Accessed July 29, 2021).

[57] Risser J Jacobson TA Kripalani S. Development and psychometric evaluation of the self-efficacy for appropriate medication use scale (SEAMS) in low-literacy patients with chronic disease. J Nurs Meas. (2007) 15:203–19. doi: 10.1891/106137407783095757, PMID: 18232619

[58] Polsook R Aungsuroch Y Thanasilp S Duffy JR. Editors. Validity and reliability of Thai version of questionnaire measuring self-efficacy for appropriate medication use scale among Thai with post-myocardial infarction. Songklanakarin J Sci Technol. (2014) 36:411–7.

[59] Knobel H Alonso J Casado JL Collazos J González J Ruiz I. Validation of a simplified medication adherence questionnaire in a large cohort of HIV-infected patients: the GEEMA study. AIDS. (2002) 16:605–13. doi: 10.1097/00002030-200203080-00012, PMID: 11873004

[60] Arenas MD Malek T Álvarez-Ude F Gil MT Moledous A Reig-Ferrer A. Phosphorus binders: preferences of patients on haemodialysis and its impact on treatment compliance and phosphorus control. Nefrologia. (2010) 30:522–30. doi: 10.3265/Nefrologia.pre2010.may.10275, PMID: 20613851

[61] Laranjo L. (2016). “Participatory health through social media,’’ in Chapter 6 - Social media and health behavior change. eds. S. Syed-Abdul, E. Gabarron, and A. Y. S. Lau (Academic Press), 83–111.

[62] Linden A Biuso T Butterworth S. Help patients with chronic kidney disease stave off dialysis. J Fam Pract. (2010) 59:212–9. PMID: 20398579

[63] Bischof G Bischof A Rumpf HJ. Motivational interviewing: an evidence-based approach for use in medical practice. Dtsch Arztebl Int. (2021) 118:109–15. doi: 10.3238/arztebl.m2021.0014, PMID: 33835006 PMC8200683

[64] Rollnick S Miller W. What is motivational interviewing? Behav Cogn Psychother. (1995) 23:325–34. doi: 10.1017/S135246580001643X, PMID: 19364414

[65] Peters M Potter CM Kelly L Fitzpatrick R. Self-efficacy and health-related quality of life: a cross-sectional study of primary care patients with multi-morbidity. Health Qual Life Outcomes. (2019) 17:37. doi: 10.1186/s12955-019-1103-3, PMID: 30764833 PMC6376655

[66] Ghisi GLM Aultman C Vanzella L Konidis R Sandison N Oh P. Effectiveness of a virtual vs. in-person group-based education curriculum to increase disease-related knowledge and change health behaviour among cardiac rehabilitation participants. Patient Educ Couns. (2023) 118:108021. doi: 10.1016/j.pec.2023.108021, PMID: 37866071

[67] Vrasidas C McIsaac MS. Principles of pedagogy and evaluation for web-based learning. Int J Phytoremediation. (2000) 21:105–11. doi: 10.1080/095239800410405

[68] Lambert K Mullan J Mansfield K. An integrative review of the methodology and findings regarding dietary adherence in end stage kidney disease. BMC Nephrol. (2017) 18:318. doi: 10.1186/s12882-017-0734-z 29061163 PMC5653982

[69] Kugler C Maeding I Russell CL. Non-adherence in patients on chronic hemodialysis: an international comparison study. J Nephrol. (2011) 24:366–75. doi: 10.5301/JN.2010.5823, PMID: 20954134

[70] Kugler C Vlaminck H Haverich A Maes B. Nonadherence with diet and fluid restrictions among adults having hemodialysis. J Nurs Scholarsh. (2005) 37:25–9. doi: 10.1111/j.1547-5069.2005.00009.x, PMID: 15813583

[71] Ozen N Cinar FI Askin D Mut D Turker T. Nonadherence in hemodialysis patients and related factors: a multicenter study. J Nurs Res. (2019) 27:e36. doi: 10.1097/jnr.0000000000000309, PMID: 30720548 PMC6641098

